# Understanding motivations behind medical student involvement in COVID-19 pandemic relief efforts

**DOI:** 10.1186/s12909-022-03900-y

**Published:** 2022-12-05

**Authors:** Tiffany R. Bellomo, Sanjana Prasad, Deesha Bhaumik, Julia Cartwright, Yibing Zhang, Lyna Azzouz, Christian Vercler

**Affiliations:** 1 Center for Bioethics and Social Sciences in Medicine, University of Michigan Medical School, Ann Arbor, MI USA; 2 School of Public Health, University of Michigan, Ann Arbor, MI 48105 USA; 3 Section of Plastic Surgery, Department of Surgery, University of Michigan Medical School, Mott Craniofacial Offices, 4Th Floor, 1540 E. Medical Center Drive, Ann Arbor, MI 48109 USA

**Keywords:** COVID-19, SARS-CoV-2, Medical student volunteer, Ethics, Pandemic relief efforts

## Abstract

**Background:**

Although students were removed from patient-facing settings at the beginning of the COVID-19 pandemic due to concerns of burdening teaching physicians and depleting personal protective equipment, some leaders suggest students can be effectively utilized when personnel resources may be scarce. There have been narrative discussions surrounding medical student involvement, but no studies exploring the attitudes of these students. The authors aim to quantify the degree to which factors influenced a medical student’s decision to or to not volunteer during the pandemic and to characterize medical students’ attitudes towards medical professionals’ duty to serve in a pandemic.

**Methods:**

The authors developed and tested a secure web-based survey before distribution to students at 23 different US allopathic medical schools that did not graduate medical students early to aid in pandemic efforts between April and June 2020. Of the 599 students who completed the survey, 65.5% self-identified as female and were on average 25.94 years old (SD = 2.5). Multiple comparisons were made based on volunteer status. Ordinal scale questions were compared with the Mann Whitney U test, and the Chi-Squared test was used for categorical variables using R version 3.62.

**Results:**

67.6% of students volunteered in pandemic relief activities and a majority of those students volunteered in non-patient-facing roles. Community service, new skills, and time commitment were top 3 influencing factors for students who volunteered, while risk to other, time commitment, and risk to self were top 3 influencing factors for students who chose not to volunteer. Compared to other specialties, students interested in primary care specialties agreed to a greater degree that physicians have a duty to serve in pandemic relief efforts.

**Conclusions:**

Medical students who volunteered cited self-serving factors and altruistic values as significant motivators. Students who did not volunteer were significantly more concerned with risks of COVID-19 exposure. However, medical students in general agreed that students should be allowed to volunteer in COVID-19 related relief efforts. As large areas of the United States continue to experience increases in COVID-19 cases, institutions should involve medical students in balancing the level of acceptable risk with the educational benefits.

**Supplementary Information:**

The online version contains supplementary material available at 10.1186/s12909-022-03900-y.

## Background

Given the increased stress healthcare systems have faced from the COVID-19 pandemic, countries worldwide are facing the question of how to best utilize their resources, including personnel. Some medical schools in the UK and US graduated final year medical students early in an effort to alleviate the healthcare burden [1–3]. However, the role of other clinical and preclinical medical students in the pandemic efforts is unclear [1, 4]. Some have argued that medical students are not prepared or obligated to accept personal risk in order to contribute to patient care, even under extraordinary circumstances. Conversely, others believe that medical students have a duty to contribute to the response efforts [5].

The Association of American Medical Colleges (AAMC) initially endorsed medical student clinical involvement, but subsequently issued a statement on March 17th advocating for removal of all students from clinical settings [5, 6]. This has been argued as necessary: medical students deplete personal protective equipment (PPE), may function as vectors of disease, and place additional burden on teaching physicians [1, 7–9]. Additionally, in the face of cancelled surgeries and medical appointments, the environment may have diminished educational value [4, 8]. There has been controversy surrounding the AAMC guidelines, as some argue trainees stand to learn a tremendous amount while mitigating workforce shortages [9, 10]. It has been suggested that medical students can assist with outpatient care, non-COVID-19 inpatient care, and even respiratory therapy [4, 9]. Outside of patient care, students have organized efforts to assist the community through providing childcare, grocery pick up, PPE collection drives, and public health department efforts [4, 5, 8, 11].

The AAMC instructed that any medical student involvement in patient-facing activities should be voluntary [9]. Interestingly, the surge of volunteerism from medical students was expected by some who note students are intrinsically motivated by a strong sense of altruism [5, 12]. Others note that volunteers were motivated by concern about lower grades, exclusion from future research opportunities, and even a sense of coercion [7]. Although narrative publications have discussed the ethical implications of medical student involvement in the pandemic, there have been no studies exploring the motivation behind medical student volunteering. Understanding motivations and attitudes toward volunteering may not only provide an ethical framework in which to discuss medical student involvement in the pandemic, but also help inform future guidelines for medical student involvement in extraordinary situations. Our aims were to identify and quantify the degree to which factors influence a student’s decision to participate in volunteer activities by different categorical measures, characterize student attitudes towards a choice or duty to serve based on volunteer status, and characterize student attitudes towards a physician’s duty to serve based on future residency type.

## Methods

All methods were carried out in accordance with relevant guidelines and regulations. This research study was reviewed by both the University of Michigan ethics committee and the University of Michigan Institutional Review Board for Human Subjects Research. These aforementioned reviewers determined this study to be exempt under the University of Michigan Institutional Review Board for Human Subjects Research #HUM00181078 and waived the need for informed consent. Although formal informed consent was not required due to the nature of this study, the first page of the survey contained general information about the survey, a statement that participation is voluntary, and a description of measures of confidentiality (Supplemental Figure 1).

### Student recruitment

Medical school student listserv administrators from 35 institutions were contacted via email between April and June 2020. The email included a detailed explanation of the study and a request to forward an email explaining how to voluntarily participate in the anonymous survey to the student listserv. Email information collected independently of survey responses would only be collected if the participant elected to receive a $10 Amazon gift card, which was offered to the first 100 participants as an incentive. After a medical school listserv administrator granted permission, a member of the study team or the listserv administrator emailed the medical student listserv the information on how to voluntarily participate in the anonymous survey. Twenty-three institutions distributed the survey to their medical school students (Supplemental Table 1).

### Survey creation

An anonymous online survey was developed using REDCap (2011), a secure, web-based survey application (Harris et al. 2009; REDCap Survey, Nashville, TN). The survey assessed the ways in which medical students were participating in COVID-19 pandemic relief efforts and their motivations. The survey was presented at the University of Michigan Center for Bioethics and Social Sciences in Medicine working group meeting, where specific feedback was provided on its content and format. The revised survey was then administered to 15 University of Michigan Medical School students who provided feedback on the clarity and readability of the questions. After another revision, the final survey (Supplemental Figure 1) was created and distributed to 23 schools (Supplemental Table 1).

### Survey topics

Survey questions included demographic questions (age, gender, year of medical school), regarding past professional experience and future specialty choice, multiple choice (yes/no) questions regarding specific volunteer involvement, Likert scale 1 to 5 questions with 0 or 6 being does not apply, and questions rating factors in the participant’s decision to volunteer in relief efforts. Students were asked to rate a series of statements on a Likert scale (0 being NA, 1 being not a factor at all, and 5 being a primary factor) related to hypothesis-driven motivations concerning the decision to or to not volunteer in COVID-19 pandemic relief efforts. The categories of motivation were developed from previous studies that show students pursue medicine and service learning due to the influence of family and friends, job prospects, intellectual capacity, personal development, and applying new concepts [13, 14]. There have also been previous studies concerning a physician’s duty to serve [15, 16], which were adapted to develop questions concerning a medical student’s duty to serve. In order to assess medical student attitudes towards a choice or duty to serve, students were also asked to rate their agreement (1 being strongly disagree, 5 being strongly agree) with working in various risk settings during a pandemic in two stages of medical training: clinical student and physician. The statements presented to participants were: 1) a medical student with clinical experience has a duty to serve in the following roles during a pandemic, 2) a medical student with clinical experience should be allowed to serve in the following roles during a pandemic, 3) in the future I, as a board-certified physician, will have a duty to serve in the following roles during a pandemic. Students rated four risk settings for each question: in-person high risk of exposure to the pandemic infectious disease, in-person low risk of exposure to the pandemic infectious disease, in-person but non-patient care roles, and remote roles. The survey can be found in the appendix.

### Statistical analysis

Data analyses were conducted using R version 3.62 (r-project.org). Descriptive statistics were conducted using frequency, mean, and standard deviation (Table 1). For the remaining analyses, the Mann Whitney U test was used to evaluate differences in the ordinal scale questions for various comparisons of interest. Chi Squared tests were used to compare categorical variables. Statistical significance was evaluated at *p* < 0.05.

**Table 1:** Baseline demographic information and COVID-19 student participation information and characteristics of study participants (*N* = 599)

	N (%)
Age (mean (SD))	25.9 (2.5)
Gender Identity	
Male	201 (33.8)
Female	389 (65.5)
Non-binary	4 (0.7)
Year of School	
Medical School Year 1	117 (19.5)
Medical School Year 2	135 (22.5)
Medical School Year 3	181 (30.2)
Medical School Year 4	131 (21.9)
Medical Science Training Program	24 (4.0)
Oral and Maxillofacial Surgery	2 (0.3)
Leave Of Absence	9 (1.5)
Core Clerkship Status	
Completed	255 (42.6)
Not Completed	205 (34.2)
Currently On	139 (23.2)
Required Participation	
Yes	21 (3.5)
No	578 (96.5)
Volunteer Status	
Yes	405 (67.6)
No	176 (29.4)
No Opportunities Available	18 (3.0)
Patient Facing Volunteer Activity	
Yes	95 (15.9)
No	430 (71.8)
No Opportunities Available	74 (12.4)

## Results

### Volunteer activities

Table 1 shows the distribution of demographic and involvement questions. There were 599 participants in this study, 65.5% of whom self-identified as female and were an average age of 25.94 years (SD = 2.5). 42.6% had completed their core clerkships, 34.2% had not, and 23.2% were currently on core clerkships. Only 3.5% of students reported that their medical school had required them to participate in activities that aid in the COVID-19 pandemic response. Although 3.0% of all students did not have any available opportunities to volunteer, 67.6% of students indicated that they volunteered with non-curricular activities that aided in the COVID-19 pandemic response. Of these students who chose to volunteer, 79.5% of students did not work in patient-facing activities but rather chose to work remotely to support relief efforts (Supplemental Figure 2). Interestingly, of the 20.5% of students who chose to volunteer in patient-facing capacities, 44.6% were working with patients who had confirmed active COVID-19 infections.

### Motivations for volunteer activities

**Fig. 1:**
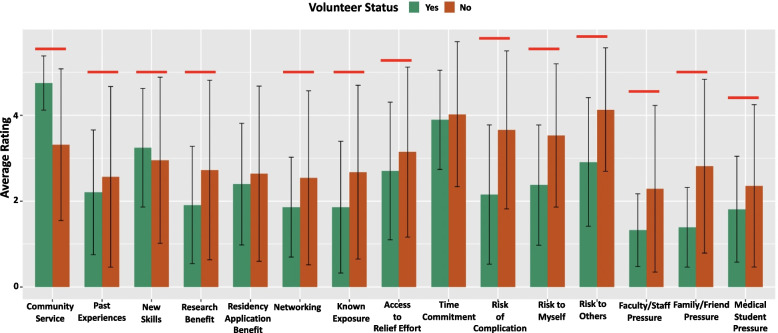
Average ratings to 15 questions pertaining to medical students’ motivation towards volunteering for COVID-19 efforts. Likert scale with 1 representing not a factor and 5 representing a primary factor in the decision to volunteer. Red brackets signify a statistically significant difference (*p* < 0.05) between the volunteer statuses. Medical students who indicated there were opportunities available to volunteer (*n* = 581)

Results of students’ rating of a series of statements related to hypothesis-driven motivations concerning the decision to or to not volunteer in COVID-19 pandemic relief efforts can be seen in Fig. 1.

When the data is stratified by volunteer status of the student, students who volunteered indicated community service (mean = 4.75 [SD = 0.63], *p* < 0.001) and gaining new skills (mean = 3.24 [SD = 1.38], *p* = 0.007) were significantly greater factors in their decision than students who did not volunteer. Students who volunteered felt on average minimal pressure from faculty/staff, family/friends, and other medical school students (faculty/staff pressure: mean = 1.32 [SD = 0.85], *p* < 0.001; family/friends pressure: mean = 1.39 [SD = 0.93], *p* < 0.001; other medical school students pressure: mean = 1.81 [SD = 1.23], *p* < 0.008).

Students who did not volunteer indicated lack of research benefit, access to the relief effort, and COVID-19 related risks were significantly greater factors in their decision than students who volunteered (research benefit: mean = 2.72 [SD = 2.09], *p* < 0.001; access to the relief effort: mean = 3.14 [SD = 1.98], *p* = 0.016; risk of COVID-19 complication: mean = 3.66 [SD = 1.84], *p* =  < 0.001; risk of COVID-19 to myself: mean = 3.52 [SD = 1.67], *p* < 0.001; risk of COVID-19 to others: mean = 4.13 [SD = 1.44], *p* < 0.001).

**Fig. 2:**
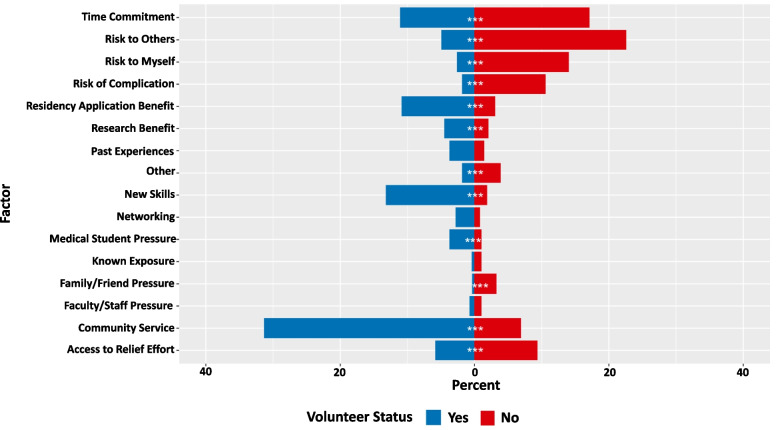
Frequency of top ranked motivation factors among students that volunteered and did not volunteer. Students were asked to rank their top 3 factors in their volunteer decision. Blue: Students who chose to volunteer in COVID-19 response initiatives. Stars indicate statistically significant differences among those that volunteered and did not volunteer (*p* < 0.05, Chi-Squared test). Red: Students who chose not to volunteer in COVID-19 response initiatives. Cohort of medical students who indicated there were opportunities available to volunteer (*n* = 581)

Students who volunteered most frequently rated community service, new skills, and time commitment as a top 3 primary factor in their decision, while students who did not volunteer most frequently rated risk to others, time commitment, and risk to myself as a top 3 primary factor in their decision (Fig. 2).

### Duty to serve

**Fig. 3:**
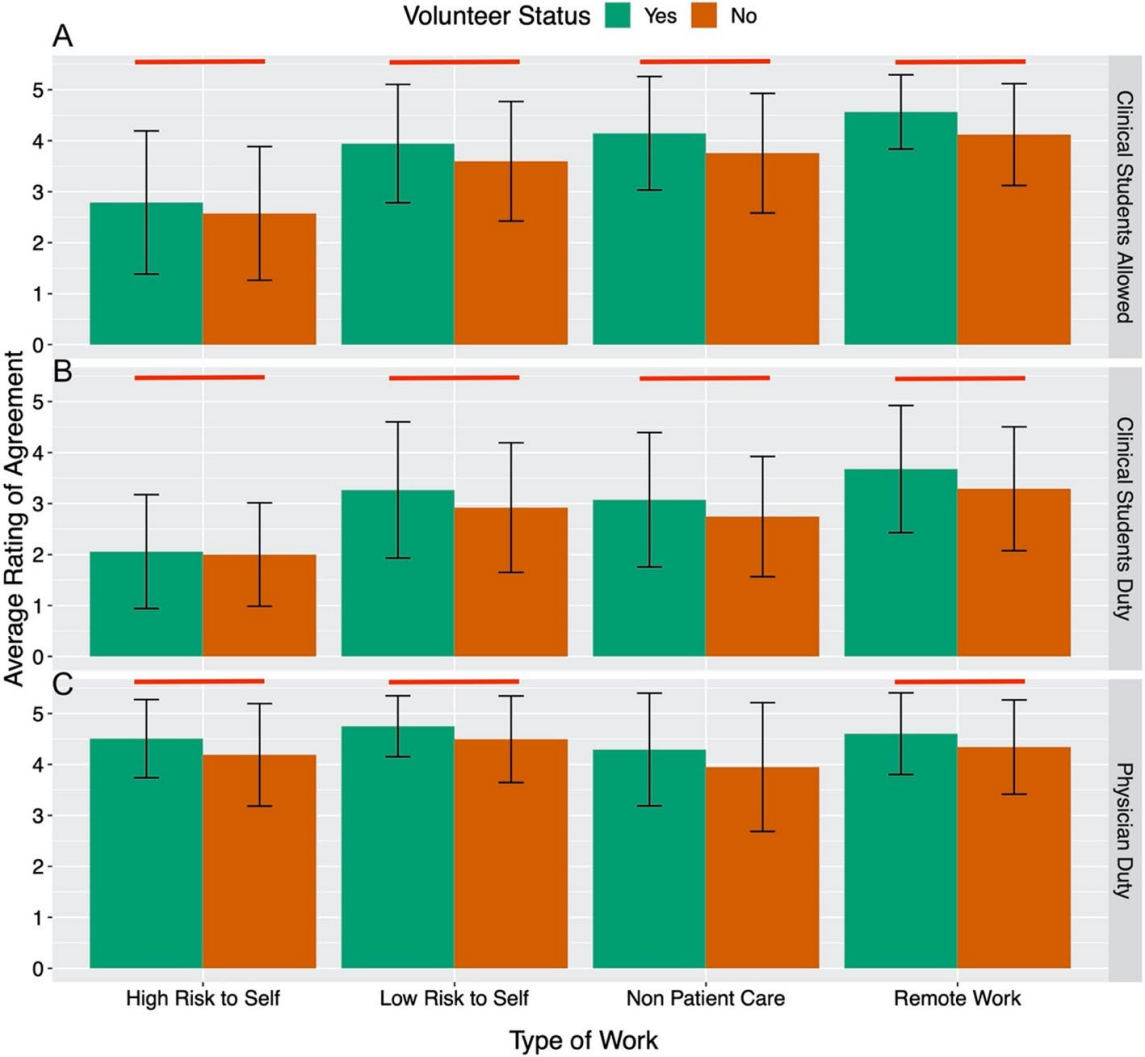
Medical students’ average rating of agreement with working in various risk settings during a pandemic based on stage of medical training (**A**-**C**). Stratified by whether or not student volunteered during COVID-19 pandemic. Likert scale with 1 representing strongly disagreeing and 5 representing strongly agreeing with working in various risk settings. Red brackets signify a statistically significant difference (*p* < 0.05) between the volunteer statuses. **A** Should Clinical students be allowed to work in these settings? **B** Do clinical students have a duty to work in these settings? **C** Do physicians have a duty to work in these settings?

**Fig. 4:**
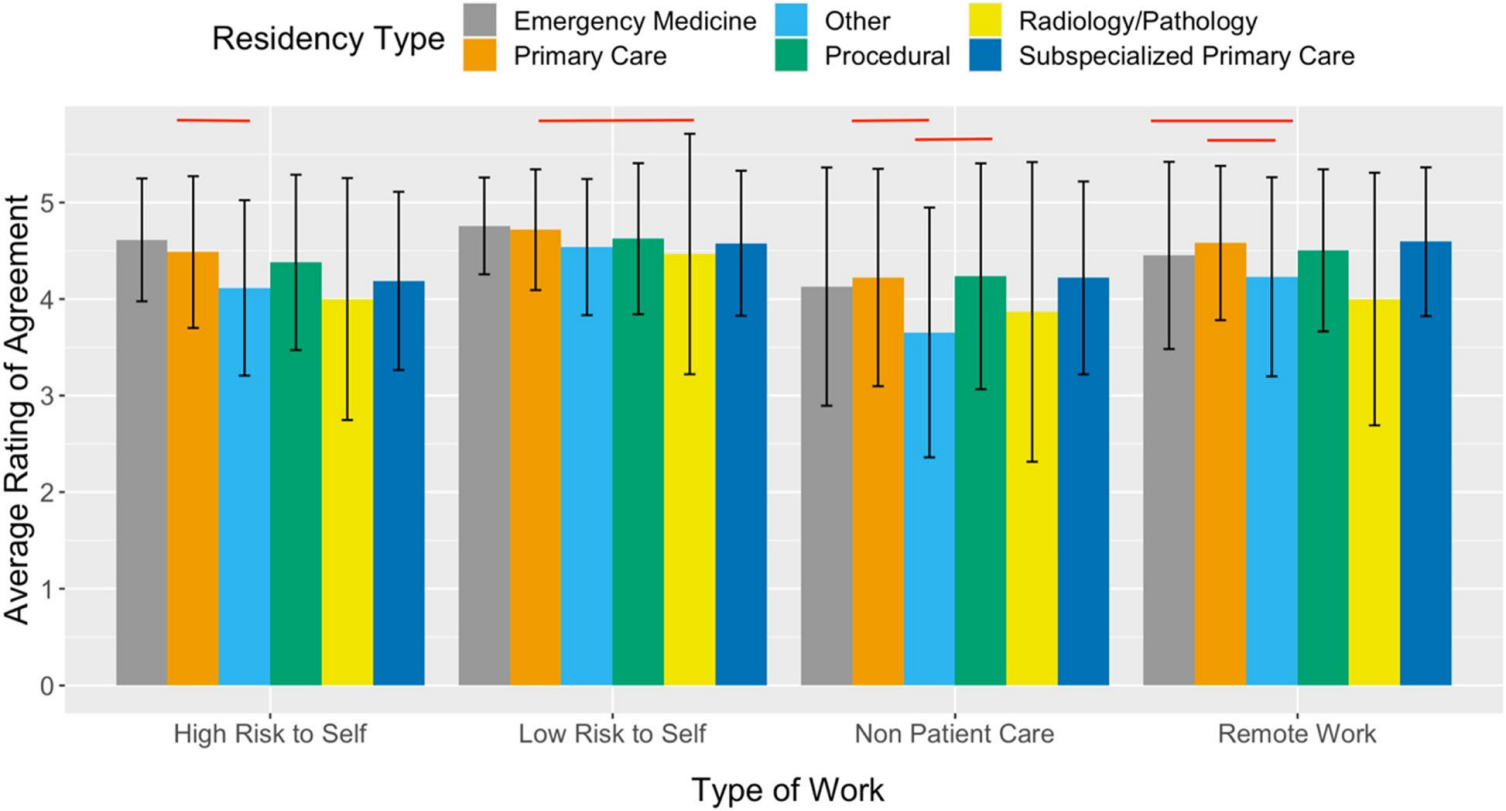
Medical students’ average rating of agreement with whether physicians have a duty to work in various risk settings during a pandemic. Stratified by student’s self selected future residency type. Likert scale with 1 representing strongly disagreeing and 5 representing strongly agreeing with working in various risk settings. Red brackets signify a statistically significant difference (*p* < 0.05) between the residency type

Figure 3 shows the results from students’ attitudes towards a choice or duty to serve in various risk settings during a pandemic as clinical students and physicians. Students who volunteered had a statistically significant higher average rating of agreement with the statement that clinical students should have the option to work in in settings of all risk levels (in-person patient care roles with a high risk of disease exposure, in-person patient care roles with a low risk of disease exposure, in-person non-patient care roles, and remote roles) when compared to students who did not volunteer (Mean [SD]; high risk to self: 2.79 [1.41] vs 2.57 [1.31]; low risk to self: 3.94 [1.16] vs 3.60 [1.17]; non-patient care: 4.15 [1.11] vs 3.76 [1.17]; remote work: 4.57 [SD = 0.73] vs 4.12 [1.00], *p* < 0.01 for all comparisons). Interestingly, students who volunteered also had a higher average rating of agreement with the statement that clinical students have a duty to work in in-person patient care roles with a high risk of disease exposure, in-person with low risk of disease exposure and in remote roles compared to students who did not volunteer (Mean [SD]; high risk to self: 2.06 [1.12] vs 2.0 [1.01], *p* = 0.01; low risk to self: 3.27 [1.33] vs 2.92 [1.27], *p* = 0.04; remote work: 3.68 [1.25] vs 3.29 [1.21], *p* = 0.002). Further, students who volunteered had a higher average rating of agreement with the statement that they as future physicians have a duty to serve in settings of all risk levels compared to students who did not volunteer (Mean [SD]; high risk to self: 4.51 [0.77] vs 4.19 [1.01]; low risk to self: 4.75 [0.60] vs 4.50 [0.85]; non patient care: 4.29 [1.11] vs 3.95 [1.26]; remote work: 4.60 [0.80] vs 4.34 [0.92], *p* < 0.01 for all comparisons). Students interested in primary care specialties agreed to a greater extent that physicians have a duty to serve in remote settings, non patient care settings, and high risk settings compared to other specialties. Students interested in primary care specialties also agreed to a greater extent that physicians have a duty to serve in low-risk settings compared to students interested in radiology/pathology specialties. Lastly, students interested in emergency medicine specialty agreed to a higher extent that physicians have a duty to conduct remote work when compared to other specialties (*p* < 0.05 for all comparisons) (Fig. 4).

## Discussion

### Motivation for volunteering

Our study found that a particular medical student’s motivation for volunteering during the COVID-19 pandemic is a combination of altruism, available time, self-serving nature, and external pressures. In our study, 67.6% of medical students volunteered of their own volition without school-required participation in such efforts. Generally, medical school recruitment is contingent upon prospective students demonstrating altruism [17]. Similarly, our findings suggest that altruism is a key motivator in students who volunteered, with 31.38% indicating serving the community as one of the three primary factors in their decision to volunteer (Fig. 2). Additionally, students who volunteered had statistically higher interest in serving the community than those who did not volunteer.

A sense of obligation to community is not the only motivation for students to volunteer. Self-serving motivators, including additional research opportunities, networking, and benefit to residency applications, account for almost one fifth of the key factors that influence a student’s decision to volunteer. Interestingly, these motivations may explain the drastic difference in volunteering participation between third-year medical students, who experience the added pressure of residency applications, and fourth-year medical students, who have already secured residency positions: 80.7% of third-year medical students in the study cohort volunteered while only 53.4% of fourth-year medical students volunteered (Supplemental Table 2). While both cohorts have substantial clinical training compared to their first- or second-year counterparts, their priorities may vary: the COVID-19 pandemic serves as an extraordinary opportunity for students to boost their curriculum vitae before applying to residency.

Extrinsic pressures to volunteer may result from fellow medical students, faculty, hospital staff, friends, and family members. These pressures were the least influential in a student’s decision to volunteer (Fig. 1); however, pressure from other medical students had a higher impact than from friends, family, faculty, or hospital staff. At most medical schools, volunteering efforts during COVID-19 were spearheaded by medical students [5]. Thus, medical students may feel the greatest pressure to volunteer from their peers.

Time commitment is an important, yet often overlooked, aspect of volunteering. Interestingly, we found that 11.1% of students who volunteered and 17.11% of students who did not volunteer indicated that this was among the top 3 primary factors in their decision (Fig. 2). In this survey, the time commitment variable could be understood as either having enough time or not having enough time for volunteer activities. With educational and clinical obligations, medical students are often limited on time for extracurricular activities and must consider opportunity costs in volunteering. Hours spent volunteering are fewer hours spent on academic development, including studying, research, and extracurricular activities. However, some students may view the time commitment as a necessary cost if they prioritize service or the potential leadership opportunities involved.

A student’s self-selected specialty may reflect their obligation to serve and moral agency. Other studies have found that medical students with a lower degree of altruism are significantly more likely to choose high-income specialties [18]. When looking at a student’s self-selected specialty in our study, primary care specialties agreed to a greater extent that physicians have a duty to serve in low risk settings and remote settings than radiology/pathology and other specialties respectively (Fig. 4). Overall, altruism may be the main motivation behind student participation in COVID-19 relief efforts, but self-serving motivations are an important secondary consideration.

### Assumption of risk by students

The AAMC released multiple guidance documents recommending that, where feasible, medical students should be excluded from direct patient care activities for a period of time while medical schools developed plans to safely re-integrate medical students into the clinical space [18]. However, even voluntary COVID-19 pandemic relief efforts that do not require interface with patients ask participants to assume some risk. In our study, students who chose not to volunteer considered risk of exposure to themselves or others as significantly more important factors in their decision than students who did volunteer (Fig. 1)**.** The AAMC identified the following as barriers to reintegrating students in clinical settings: health insurance status, testing availability, and inadequate personal protective equipment (PPE). These same barriers may also deter students from volunteering in non-clinical settings. For example, medical trainees in the United States have an expectation that appropriate PPE will be available. A primary concern at the onset of the pandemic was PPE scarcity in all settings, as inadequate PPE exposes medical students to heightened risk of exposure to a range of infectious diseases. Similarly, the lack of appropriate masks when delivering groceries or collecting PPE may have also been influences on students’ decisions not to volunteer.

Even with appropriate PPE, there is still a risk of serious illness or death. In April 2020, the Centers for Disease Control estimated that approximately 55% of healthcare workers who developed COVID-19 contracted the virus at the hospital. The median age of our cohort is 25.94 (Table 1), which falls within an age group that carries up to 6.4% risk of hospitalization and up to 3% risk of death related to COVID-19 [19]. In addition to the risk of significant bodily harm students may assume in volunteering in patient-facing roles during the pandemic, hospitalization or other exclusion from the clinical environment due to COVID-19 may delay a student’s ability to enter clinical rotations, obtain licensure, or even graduate from medical school.

### Guidelines for future student involvement

Regardless of whether or not students volunteered in COVID-19 pandemic relief efforts, this cohort of medical students largely believe that clinical students should have the opportunity to volunteer in such efforts. Institutions may utilize the data collected through this study to integrate student perspectives into future medical trainee involvement in pandemic response initiatives. Initiatives which require student presence in the hospital and regular contact with SARS-CoV-2 positive patients should consider balancing educational benefit with trainee risk exposure. Such initiatives should acknowledge the real and perceived pressures felt by medical students in both voluntary and required clinical experiences. Efforts should be made to identify risk mitigation strategies, reinforce institutional non-retaliation policies to protect trainees, and support trainees who require testing, medical care, or extended medical leave due to SARS-CoV-2 infection.

### Future directions

Our survey was distributed to students primarily in May 2020, when medical students were not in the clinical environment. By the end of July 2020, 68% of medical schools returned students to clinical clerkships [20]. Now that students are required to return to clinical spaces during a pandemic, their attitudes around serving patients, either in patient-facing or virtual roles, may be shifting and warrant further study. This survey left the interpretation of “risk” up to the student. The reason for this ambiguity is that there are many different situations that could be classified as high risk for a student. There was no objective data at the time of survey administration concerning transmission risk in hospitals. It would be very difficult to present students with a single objective risk situation and therefore interpretation of “risk” was left to be defined by the reader. Exploring further what trainees and institutions deem “acceptable” risk assumption in the course of medical training is important when considering a student’s duty to serve. As large areas of the United States continue to experience dramatic increases in COVID-19 cases, [21] this study is important for directing future involvement of medical trainees in COVID-19 response initiatives.

### Limitations

One of the main limitations of this study is concerning survey development, as there is a paucity of literature on medical student motivations for perusing a medical career and attitudes towards service. Therefore, we relied heavily on the experience of the authors and 15 students who took the piolet survey to provide feedback on survey material, which was not directly adapted from a validated survey tool. The survey was not formally tested with Cronbachs alpha or another statistical method before administration, but the survey was reviewed at a professional committee meeting and piloted by 15 medical students before release. Within the survey, the interpretation of high risk was left up to each individual student and therefore compromises the validity of the data collected. Other limitations include the small sample of medical schools to whom our survey was distributed: 23 of the 154 accredited US medical schools received our survey [22, 23]. Only 18 students who responded to the survey indicated that they had no opportunities available to volunteer in COVID-19 pandemic relief efforts, which likely does not represent the true proportion of students with this limitation. A few survey responders listed more than three factors in their indication of the top three factors that influenced their decision to volunteer or to not volunteer, and these answers were omitted from our final data analysis.

## Conclusions

Overall, we found that there were different motivating factors for students who decided to volunteer compared to students who did not: community service, new skills, and time commitment were primary factors for students who volunteered, while risk to other, time commitment, and risk to self were primary factors for students who did not volunteer. We also found that motivations for volunteering may change over the course of medical training: three-fourths of third-year medical students and half of fourth-year medical students volunteered for COVID-19 related relief efforts. Compared to students interested in radiology/pathology and other specialties, students interested in primary care specialties agreed to a greater extent that physicians have a duty to serve in low risk settings and remote settings. However, medical students in general agreed that students should be allowed to volunteer in COVID-19 related relief efforts. As large areas of the United States continue to experience increases in COVID-19 cases, institutions should involve medical students in evaluating what the acceptable risks are compared to the ethical educational benefits off student involvement in patient care.

## Supplementary Information

**Additional file 1: Supplemental Figure 1.** Complete REDCap survey distributed to medical students.

**Additional file 2: Supplemental Figure 2.** Patient populations students encountered for those who chose to volunteer. 405 medical students that volunteered in COVID-19 response initiatives.

**Additional file 3: Supplemental Figure 3.** Box and whisker plots of medical students’ median rating of agreement with working in various risk settings during a pandemic based on stage of medical training (A-C). Stratified by whether or not student volunteered during COVID-19 pandemic. Likert scale with 1 representing strongly disagreeing and 5 representing strongly agreeing with working in various risk settings. A. Should Clinical students be allowed to work in these settings? B. Do clinical students have a duty to work in these settings? C. Do physicians have a duty to work in these settings?

**Additional file 4: Supplemental Figure 4.** Box and whisker plot of medical students’ rating of agreement with whether physicians have a duty to work in various risk settings during a pandemic. Stratified by student’s self selected future residency type. Likert scale with 1 representing strongly disagreeing and 5 representing strongly agreeing with working in various risk settings.

**Additional file 5: Supplemental Table 1.** Participating institutions that distributed the survey to their medical students.

**Additional file 6: Supplemental Table 2.** Selected baseline medical school characteristics of study cohort stratified by volunteer status (N = 599).

**Additional file 7: Supplemental Table 3.** Raw data collected from survery used for analysis.

## Data Availability

All data generated or analyzed during this study are included in this published article (Supplemental Figure 1 and Supplemental Table 3).

## Abbreviations

AAMC: The association of american medical colleges
PPE: Personal protective equipment
