# Supplementary material for: Understanding motivations behind medical student involvement in COVID-19 pandemic relief efforts
Source: BMC Med Educ. 2022 Dec 5;22:837. doi: 10.1186/s12909-022-03900-y (PMC9721039; doi:10.1186/s12909-022-03900-y)
Supplement: Supplementary file 1 — Additional file 1: Supplemental Figure 1. Complete REDCap survey distributed to medical students. [file 12909_2022_3900_MOESM1_ESM.docx]

**Supplemental Figure 1.** Complete REDCap survey distributed to medical students.

| Do you attend an allopathic medical school in the United States of America? | radio, Required   \| 1 \| Yes \| \| --- \| --- \| \| 2 \| No \| |
| --- | --- | --- | --- | --- | --- |
| Is your medical school graduating students early to aid in COVID19 response? | radio, Required   \| 1 \| Yes \| \| --- \| --- \| \| 2 \| No \| |
| Section Header: *Please answer the following questions* | |
| Does your medical school require you to participate in activities that aid in the COVID-19 pandemic response? | radio, Required   \| 1 \| Yes \| \| --- \| --- \| \| 2 \| No \| |
| Are you involved with COVID-19 response initiatives that require you to work in person with patients? | radio, Required   \| 1 \| Yes \| \| --- \| --- \| \| 2 \| No \| \| 3 \| No opportunities available \| |
| What patient population are you working with? Check all that apply. | checkbox   \| 1 \| Patients with confirmed or suspected active COVID-19 infection \| \| --- \| --- \| \| 2 \| Patients without suspected COVID-19 infection \| \| 3 \| Patients with resolved COVID-19 infection \| |
| Section Header:  Did you decide to volunteer with any non-curricular volunteer activities that aid in the COVID-19 pandemic response? | radio, Required   \| 1 \| Yes \| \| --- \| --- \| \| 2 \| No \| \| 3 \| No opportunities available \| |
| Keeping in mind your response to the previous question, rate the degree to which the following factors influenced your decision on a scale of 1-5 (1 being not a factor at all to 5 being a primary factor, N/A = Not Applicable) | |
| The possibility of publishing papers related to my COVID response activities, or presenting this work at a conference | radio (Matrix), Required   \| 1 \| 1 - Not a factor at all \| \| --- \| --- \| \| 2 \| 2 \| \| 3 \| 3 \| \| 4 \| 4 \| \| 5 \| 5 - Primary factor \| \| 6 \| N/A \| |
| Pressure from faculty and staff at my home institution | radio (Matrix), Required   \| 1 \| 1 - Not a factor at all \| \| --- \| --- \| \| 2 \| 2 \| \| 3 \| 3 \| \| 4 \| 4 \| \| 5 \| 5 - Primary factor \| \| 6 \| N/A \| |
| Interest in serving the community | radio (Matrix), Required   \| 1 \| 1 - Not a factor at all \| \| --- \| --- \| \| 2 \| 2 \| \| 3 \| 3 \| \| 4 \| 4 \| \| 5 \| 5 - Primary factor \| \| 6 \| N/A \| |
| Interest in acquiring new skills afforded by volunteer opportunities | radio (Matrix), Required   \| 1 \| 1 - Not a factor at all \| \| --- \| --- \| \| 2 \| 2 \| \| 3 \| 3 \| \| 4 \| 4 \| \| 5 \| 5 - Primary factor \| \| 6 \| N/A \| |
| Personally at risk for or live with someone who is at risk for COVID-19 complications | radio (Matrix), Required   \| 1 \| 1 - Not a factor at all \| \| --- \| --- \| \| 2 \| 2 \| \| 3 \| 3 \| \| 4 \| 4 \| \| 5 \| 5 - Primary factor \| \| 6 \| N/A \| |
| Networking afforded by volunteer opportunities | radio (Matrix), Required   \| 1 \| 1 - Not a factor at all \| \| --- \| --- \| \| 2 \| 2 \| \| 3 \| 3 \| \| 4 \| 4 \| \| 5 \| 5 - Primary factor \| \| 6 \| N/A \| |
| Pressure from friends, family, or loved ones | radio (Matrix), Required   \| 1 \| 1 - Not a factor at all \| \| --- \| --- \| \| 2 \| 2 \| \| 3 \| 3 \| \| 4 \| 4 \| \| 5 \| 5 - Primary factor \| \| 6 \| N/A \| |
| The ability to put pandemic response experiences on a residency application | radio (Matrix), Required   \| 1 \| 1 - Not a factor at all \| \| --- \| --- \| \| 2 \| 2 \| \| 3 \| 3 \| \| 4 \| 4 \| \| 5 \| 5 - Primary factor \| \| 6 \| N/A \| |
| Prior work experience or formal education | radio (Matrix), Required   \| 1 \| 1 - Not a factor at all \| \| --- \| --- \| \| 2 \| 2 \| \| 3 \| 3 \| \| 4 \| 4 \| \| 5 \| 5 - Primary factor \| \| 6 \| N/A \| |
| Not enough time to volunteer | radio (Matrix), Required   \| 1 \| 1 - Not a factor at all \| \| --- \| --- \| \| 2 \| 2 \| \| 3 \| 3 \| \| 4 \| 4 \| \| 5 \| 5 - Primary factor \| \| 6 \| N/A \| |
| The possibility of exposing myself to unnecessary risk | radio (Matrix), Required   \| 1 \| 1 - Not a factor at all \| \| --- \| --- \| \| 2 \| 2 \| \| 3 \| 3 \| \| 4 \| 4 \| \| 5 \| 5 - Primary factor \| \| 6 \| N/A \| |
| The possibility of exposing people around me to unnecessary risk | radio (Matrix), Required   \| 1 \| 1 - Not a factor at all \| \| --- \| --- \| \| 2 \| 2 \| \| 3 \| 3 \| \| 4 \| 4 \| \| 5 \| 5 - Primary factor \| \| 6 \| N/A \| |
| Personal or close contact with a suspected or confirmed COVID-19 infection | radio (Matrix), Required   \| 1 \| 1 - Not a factor at all \| \| --- \| --- \| \| 2 \| 2 \| \| 3 \| 3 \| \| 4 \| 4 \| \| 5 \| 5 - Primary factor \| \| 6 \| N/A \| |
| Geographic proximity to my medical school during the pandemic | radio (Matrix), Required   \| 1 \| 1 - Not a factor at all \| \| --- \| --- \| \| 2 \| 2 \| \| 3 \| 3 \| \| 4 \| 4 \| \| 5 \| 5 - Primary factor \| \| 6 \| N/A \| |
| Pressure from medical students | radio (Matrix), Required   \| 1 \| 1 - Not a factor at all \| \| --- \| --- \| \| 2 \| 2 \| \| 3 \| 3 \| \| 4 \| 4 \| \| 5 \| 5 - Primary factor \| \| 6 \| N/A \| |
| Had enough time to volunteer | radio (Matrix), Required   \| 1 \| 1 - Not a factor at all \| \| --- \| --- \| \| 2 \| 2 \| \| 3 \| 3 \| \| 4 \| 4 \| \| 5 \| 5 - Primary factor \| \| 6 \| N/A \| |
| Section Header:  Of the choices listed in the pervious question, what were the top 3 influencing factors in your decision? (select only three choices) | checkbox, Required   \| 1 \| The possibility of publishing papers or presenting work at a conference \| \| --- \| --- \| \| 2 \| The ability to put pandemic response experiences on a residency application \| \| 3 \| Pressure from medical students \| \| 4 \| Pressure from faculty and staff at my home institution \| \| 5 \| Pressure from friends, family, or loved ones \| \| 6 \| Interest in serving the community \| \| 7 \| Time commitment \| \| 8 \| Interest in acquiring new skills afforded by volunteer opportunities \| \| 9 \| Networking afforded by volunteer opportunities \| \| 10 \| Prior work experience or formal education \| \| 11 \| The possibility of exposing myself to unnecessary risk \| \| 12 \| The possibility of exposing people around me to unnecessary risk \| \| 13 \| Personally at risk for or live with someone who is at risk for COVID-19 complications \| \| 14 \| Personal or close contact with a suspected or confirmed COVID-19 infection \| \| 15 \| Geographic proximity to my medical school during the pandemic \| \| 16 \| Other \| |
| If you selected "other", please specify your reason here: | text |
| Section Header: *Please rate how much you agree with each statement* | |
| *A medical student with clinical experience has a duty to serve in the following roles during a pandemic:* | |
| In-person patient care roles with high risk of exposure to pandemic infectious disease | radio (Matrix), Required   \| 1 \| Strongly disagree \| \| --- \| --- \| \| 2 \| Somewhat disagree \| \| 3 \| Neither agree nor disagree \| \| 4 \| Somewhat agree \| \| 5 \| Strongly agree \| |
| In-person patient care roles with low risk of exposure to pandemic infectious disease | radio (Matrix), Required   \| 1 \| Strongly disagree \| \| --- \| --- \| \| 2 \| Somewhat disagree \| \| 3 \| Neither agree nor disagree \| \| 4 \| Somewhat agree \| \| 5 \| Strongly agree \| |
| In-person non-patient care roles | radio (Matrix), Required   \| 1 \| Strongly disagree \| \| --- \| --- \| \| 2 \| Somewhat disagree \| \| 3 \| Neither agree nor disagree \| \| 4 \| Somewhat agree \| \| 5 \| Strongly agree \| |
| Remote roles | radio (Matrix), Required   \| 1 \| Strongly disagree \| \| --- \| --- \| \| 2 \| Somewhat disagree \| \| 3 \| Neither agree nor disagree \| \| 4 \| Somewhat agree \| \| 5 \| Strongly agree \| |
| Section Header: *Please rate how much you agree with each statement* | |
| *A medical student with clinical experience should be allowed to serve in the following roles during a pandemic:* | |
| In-person patient care roles with high risk of exposure to pandemic infectious disease | radio (Matrix), Required   \| 1 \| Strongly disagree \| \| --- \| --- \| \| 2 \| Somewhat disagree \| \| 3 \| Neither agree nor disagree \| \| 4 \| Somewhat agree \| \| 5 \| Strongly agree \| |
| In-person patient care roles with low risk of exposure to pandemic infectious disease | radio (Matrix), Required   \| 1 \| Strongly disagree \| \| --- \| --- \| \| 2 \| Somewhat disagree \| \| 3 \| Neither agree nor disagree \| \| 4 \| Somewhat agree \| \| 5 \| Strongly agree \| |
| In-person non-patient care roles | radio (Matrix), Required   \| 1 \| Strongly disagree \| \| --- \| --- \| \| 2 \| Somewhat disagree \| \| 3 \| Neither agree nor disagree \| \| 4 \| Somewhat agree \| \| 5 \| Strongly agree \| |
| Remote roles | radio (Matrix), Required   \| 1 \| Strongly disagree \| \| --- \| --- \| \| 2 \| Somewhat disagree \| \| 3 \| Neither agree nor disagree \| \| 4 \| Somewhat agree \| \| 5 \| Strongly agree \| |
| Section Header: *Please rate how much you agree with each statement* | |
| *In the future I, as a board-certified physician, will have a duty to serve in the following roles during a pandemic:* | |
| In-person patient care roles with high risk of exposure to pandemic infectious disease | radio (Matrix), Required   \| 1 \| Strongly disagree \| \| --- \| --- \| \| 2 \| Somewhat disagree \| \| 3 \| Neither agree nor disagree \| \| 4 \| Somewhat agree \| \| 5 \| Strongly agree \| |
| In-person patient care roles with low risk of exposure to pandemic infectious disease | radio (Matrix), Required   \| 1 \| Strongly disagree \| \| --- \| --- \| \| 2 \| Somewhat disagree \| \| 3 \| Neither agree nor disagree \| \| 4 \| Somewhat agree \| \| 5 \| Strongly agree \| |
| In-person non-patient care roles | radio (Matrix), Required   \| 1 \| Strongly disagree \| \| --- \| --- \| \| 2 \| Somewhat disagree \| \| 3 \| Neither agree nor disagree \| \| 4 \| Somewhat agree \| \| 5 \| Strongly agree \| |
| Remote roles | radio (Matrix), Required   \| 1 \| Strongly disagree \| \| --- \| --- \| \| 2 \| Somewhat disagree \| \| 3 \| Neither agree nor disagree \| \| 4 \| Somewhat agree \| \| 5 \| Strongly agree \| |
| Section Header: *Demographics* | |
| What is your year of medical school? | radio, Required   \| 1 \| M1 \| \| --- \| --- \| \| 2 \| M2 \| \| 3 \| M3 \| \| 4 \| M4 \| \| 5 \| Medical Scientist Training Program (MSTP) \| \| 6 \| Oral & Maxillofacial Surgery (OMFS) \| \| 7 \| Leave of Absence (LOA) \| |
| Have you completed your core clerkships? | radio, Required   \| 1 \| Yes \| \| --- \| --- \| \| 2 \| No \| \| 3 \| Currently on core clerkships \| |
| Gender Identity: | radio, Required   \| 1 \| Male \| \| --- \| --- \| \| 2 \| Female \| \| 3 \| Non-binary \| \| 4 \| Prefer to self-describe \| \| 5 \| Prefer not to answer \| |
| Self-describe gender: | text |
| What is your age in years? | dropdown, Required |
| Do you have prior work experience or formal education in any of the following areas? Check all that apply. | checkbox, Required   \| 1 \| Public health \| \| --- \| --- \| \| 2 \| Engineering \| \| 3 \| Allied health medical professional \| \| 4 \| Social work \| \| 5 \| Mental health services \| \| 6 \| Military \| \| 7 \| Teaching \| \| 8 \| Government service organization (ex, Americorps, peace corps, Teach for America) \| \| 9 \| Non-governmental organization (NGO) or non-profit \| \| 10 \| Research \| \| 11 \| Business, \| \| 12 \| Finance \| \| 13 \| None of the above \| |
| If you have prior work experience or formal education in an area not listed above, please list it here: | text |
| If you had to apply to residency now, what would be your top specialty of interest? Select one. | dropdown, Required   \| 1 \| Anesthesiology \| \| --- \| --- \| \| 2 \| Surgery (General, Neurosurgery, Colorectal, Orthopaedic, ENT, Plastic, Thoracic) \| \| 3 \| Oral & Maxillofacial Surgery \| \| 4 \| Dermatology \| \| 5 \| Emergency Medicine \| \| 6 \| Family Medicine \| \| 7 \| Internal Medicine \| \| 8 \| Obstetrics & Gynecology \| \| 9 \| Ophthalmology \| \| 10 \| Pathology \| \| 11 \| Pediatrics \| \| 13 \| Physical Medicine & Rehabilitation \| \| 14 \| Psychiatry \| \| 15 \| Neurology \| \| 16 \| Radiology \| \| 17 \| Urology \| \| 18 \| I do not plan on applying to residency \| \| 19 \| None of the above \| |
| If you selected "none of the above" please enter your specialty of choice here: | text |
| Section Header:  Thank you for taking the survey. You will now be redirected to a survey where you may choose to enter your email address for a gift card and/or to participate in future studies. This is optional and will not affect your survey results. Your email will not be linked to the responses you provided in this survey. If you do not wish to be directed to this survey, click "submit" then close your browser window. | |
| Here you may provide your email address to receive a gift card and/or to be contacted in the future for participation in focus groups or additional surveys. Your email will not be linked to your survey results. If you choose not to provide your email address, this will not affect your participation or survey results. | descriptive |
| Please enter your email address to be eligible to receive a $10 gift card. The first 100 participants will receive a gift card. This is optional, will not be linked to your survey results, and will not affect your participation in this study. | text (email), Identifier |
| By entering your email address in this box, you consent to be contacted in the future for additional surveys or participation in focus groups. This is optional, will not be linked to your survey results, and will not affect your participation in this study. | text (email), Identifier |
