# Supplementary material for: Understanding motivations behind medical student involvement in COVID-19 pandemic relief efforts
Source: BMC Med Educ. 2022 Dec 5;22:837. doi: 10.1186/s12909-022-03900-y (PMC9721039; doi:10.1186/s12909-022-03900-y)
Supplement: Supplementary file 2 — Additional file 2: Supplemental Figure 2. Patient populations students encountered for those who chose to volunteer. 405 medical students that volunteered in COVID-19 response initiatives. [file 12909_2022_3900_MOESM2_ESM.docx]

**Supplemental Figure 2.** Patient populations students encountered for those who chose to volunteer. 405 medical students that volunteered in COVID-19 response initiatives.

**
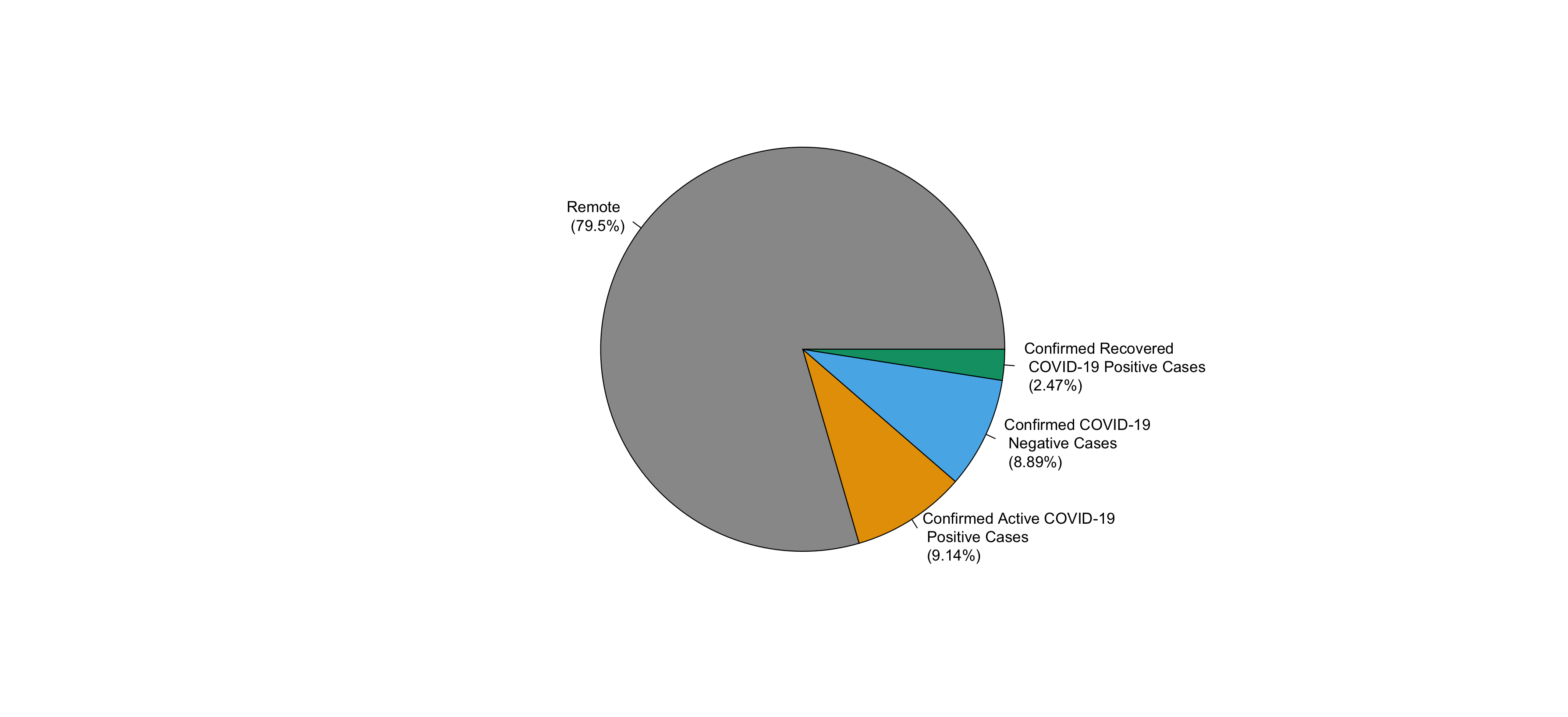
**
