# Supplementary material for: Understanding motivations behind medical student involvement in COVID-19 pandemic relief efforts
Source: BMC Med Educ. 2022 Dec 5;22:837. doi: 10.1186/s12909-022-03900-y (PMC9721039; doi:10.1186/s12909-022-03900-y)
Supplement: Supplementary file 3 — Additional file 3: Supplemental Figure 3. Box and whisker plots of medical students’ median rating of agreement with working in various risk settings during a pandemic based on stage of medical training (A-C). Stratified by whether or not student volunteered during COVID-19 pandemic. Likert scale with 1 representing strongly disagreeing and 5 representing strongly agreeing with working in various risk settings. A. Should Clinical students be allowed to work in these settings? B. Do clinical students have a duty to work in these settings? C. Do physicians have a duty to work in these settings? [file 12909_2022_3900_MOESM3_ESM.docx]

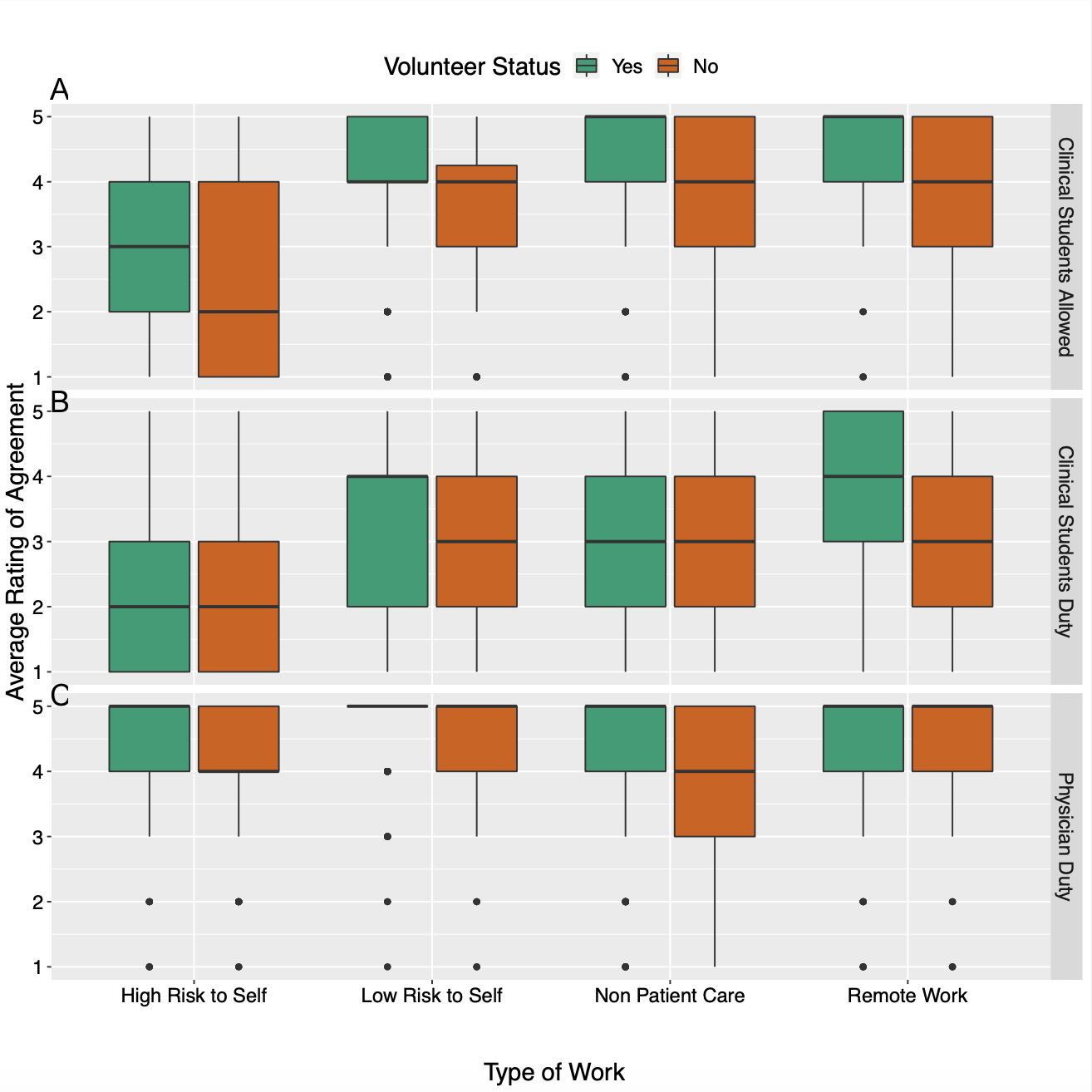


**Supplemental Figure 3.** Box and whisker plots of medical students’ median rating of agreement with working in various risk settings during a pandemic based on stage of medical training (A-C). Stratified by whether or not student volunteered during COVID-19 pandemic. Likert scale with 1 representing strongly disagreeing and 5 representing strongly agreeing with working in various risk settings. A. Should Clinical students be allowed to work in these settings? B. Do clinical students have a duty to work in these settings? C. Do physicians have a duty to work in these settings?
