# Supplementary material for: Understanding motivations behind medical student involvement in COVID-19 pandemic relief efforts
Source: BMC Med Educ. 2022 Dec 5;22:837. doi: 10.1186/s12909-022-03900-y (PMC9721039; doi:10.1186/s12909-022-03900-y)
Supplement: Supplementary file 4 — Additional file 4: Supplemental Figure 4. Box and whisker plot of medical students’ rating of agreement with whether physicians have a duty to work in various risk settings during a pandemic. Stratified by student’s self selected future residency type. Likert scale with 1 representing strongly disagreeing and 5 representing strongly agreeing with working in various risk settings. [file 12909_2022_3900_MOESM4_ESM.docx]

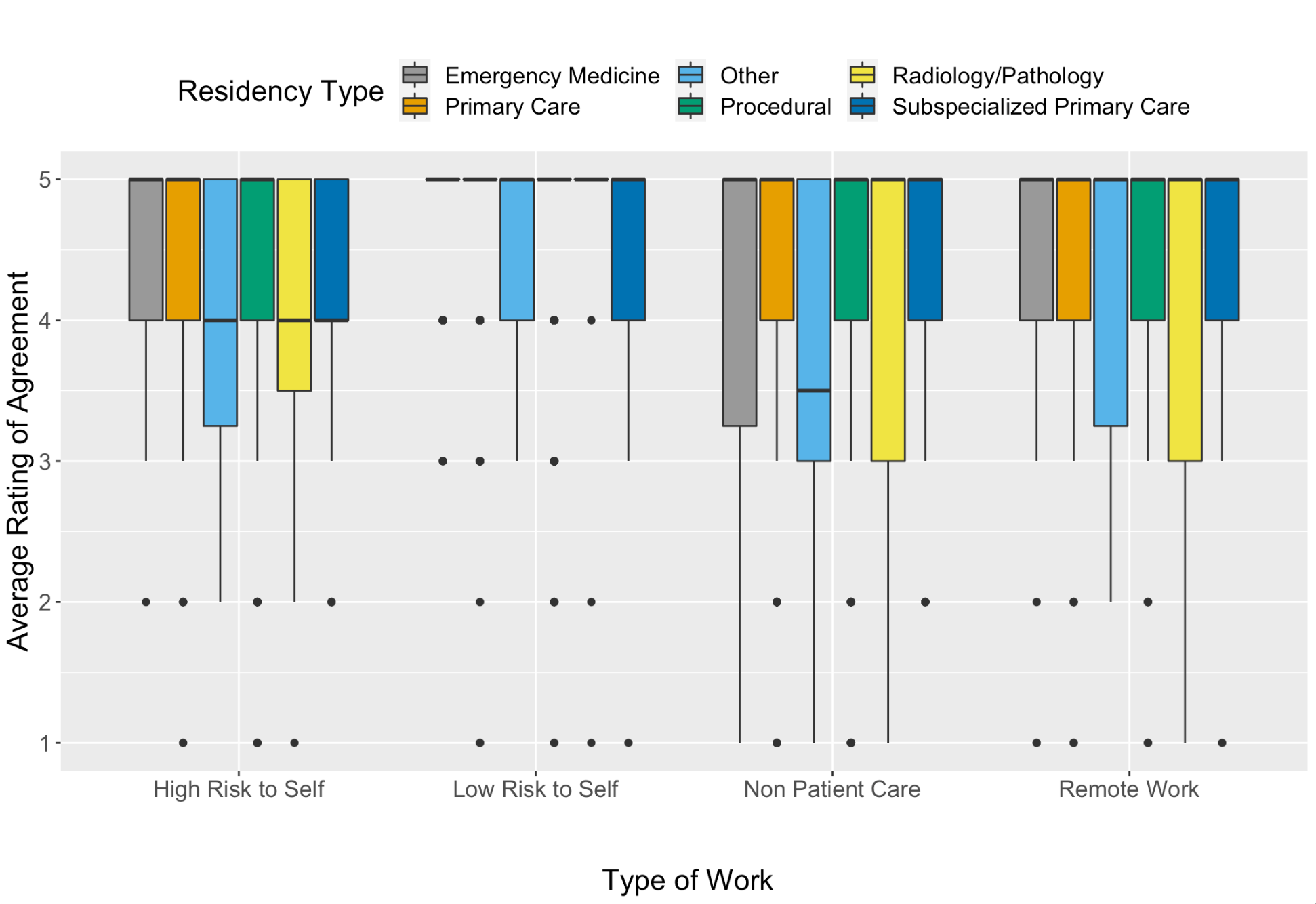


**Supplemental Figure 4.** Box and whisker plot of medical students’ rating of agreement with whether physicians have a duty to work in various risk settings during a pandemic. Stratified by student’s self selected future residency type. Likert scale with 1 representing strongly disagreeing and 5 representing strongly agreeing with working in various risk settings.
