# Supplementary material for: Understanding motivations behind medical student involvement in COVID-19 pandemic relief efforts
Source: BMC Med Educ. 2022 Dec 5;22:837. doi: 10.1186/s12909-022-03900-y (PMC9721039; doi:10.1186/s12909-022-03900-y)
Supplement: Supplementary file 5 — Additional file 5: Supplemental Table 1. Participating institutions that distributed the survey to their medical students. [file 12909_2022_3900_MOESM5_ESM.docx]

**Supplemental Table 1.** Participating institutions that distributed the survey to their medical students.

| Participating schools |
| --- |
| Cleveland clinic Lerner College of Medicine |
| Cooper Medical School of Rowan University |
| Johns Hopkins University |
| Michigan State University |
| Mississippi Medical Center |
| Northwestern University |
| Ohio State University |
| Stanford University |
| Texas A&M University |
| University of Arizona |
| University of California Davis |
| University of California Irvine |
| University of California San Diego |
| University of California San Francisco |
| University of Chicago |
| University of Maryland |
| University of Miami |
| University of Michigan |
| University of Minnesota |
| University of Pittsburgh |
| University of Southern California |
| University of Texas Southwestern |
| University of Wisconsin |
| Wayne State University |
| Zucker School of Medicine at Hofstra/NOrthwell |
