# Supplementary material for: Understanding motivations behind medical student involvement in COVID-19 pandemic relief efforts
Source: BMC Med Educ. 2022 Dec 5;22:837. doi: 10.1186/s12909-022-03900-y (PMC9721039; doi:10.1186/s12909-022-03900-y)
Supplement: Supplementary file 6 — Additional file 6: Supplemental Table 2. Selected baseline medical school characteristics of study cohort stratified by volunteer status (N = 599). [file 12909_2022_3900_MOESM6_ESM.docx]

**Supplemental Table 2.** Selected baseline medical school characteristics of study cohort stratified by volunteer status (N = 599).

|  | N | Volunteer Status N (%) | | |
| --- | --- | --- | --- | --- |
|  |  | Yes | No | No Opportunities Available |
| **Year of School/Program** |  |  |  |  |
| Medical School Year 1 | 117 | 74 (63.2) | 37 (31.6) | 6 (5.1) |
| Medical School Year 2 | 135 | 92 (68.1) | 40 (29.6) | 3 (2.2) |
| Medical School Year 3 | 181 | 146 (80.7) | 33 (18.2) | 2 (1.1) |
| Medical School Year 4 | 131 | 70 (53.4) | 55 (42.0) | 6 (4.6) |
| Medical Science Training Program | 24 | 16 (66.7) | 7 (29.2) | 1 (4.2) |
| Oral and Maxillofacial Surgery | 2 | 1 (50.0) | 1 (50.0) | 0 (0.0) |
| Leave Of Absence | 9 | 6 (66.7) | 3 (33.3) | 0 (0.0) |
| **Core Clerkship Status (%)** |  |  |  |  |
| Completed | 255 | 169 (66.3) | 80 (31.4) | 6 (2.4) |
| Not Completed | 205 | 126 (61.5) | 70 (34.1) | 9 (4.4) |
| Currently On | 139 | 110 (79.1) | 26 (18.7) | 3 (2.2) |
